# Reading Dental Radiographs

**Published:** 1918-02

**Authors:** Frank D. Price

**Affiliations:** Toronto


### READING DENTAL RADIOGRAPHS

FRANK D. PRICE, D.D.S., TORONTO

In the August *Oral Health* appeared an article beginning with this paragraph: "The time has arrived when the dental profession will have to take a decided stand upon the whole question of radiography. Under existing conditions incalculable harm is being done to the honest, conscientious 'family dentist' who looks upon radiography as a photographic side line. What do these men care whether ethics go to the winds so long as they get a substantial dividend upon their investment in a 'machine?' " Then appears more than two pages to amplify the above.

Quite naturally I resented the above very much as I was fairly corralled in his little ring of black sheep. Soon, however, resentment changed to pity, because the writer had poor arguments to support his statements, and I thought I saw between the lines an honest appeal for help in reading dental negatives.

Much might be said. It is a pity that so little time and practice has been afforded at the dental colleges to correct the above. Many less important subjects get more attention. The X-ray is scientific and a faithful revealer of bone conditions and therefore worthy of being familiar to every dental student.

Many excellent dental radiographs are made in hospitals by operators who are not dentists. These men can not be expected to correctly interpret dental negatives. The remedy should lie with the dentist. Every dentist should be able to read dental negatives. It is not often necessary, as the writer inferred, that the radiographer should know any clinical history of a particular tooth. He makes a negative picture of that tooth and the surrounding tissues. It is often as plain to read as the print on this page. Even a poor radiograph is a great help. And no magic was ever more wonderful than the marvelous revelation often afforded by a dental negative. I have seen beauty of detail unsurpassed by any photograph. I have seen the most unexpected conditions revealed, without which the tooth must have been lost or perhaps the patient's happiness, health and often life jeopardized. And the X-ray will be not less used, but will be used more as the weeks and years pass.

Perhaps just now we are looking most at the bone conditions about root apices. Spend time in examining your next good radiograph. We suppose it is a negative, not a print. A print is simpler to study; it is positive. But not all in a negative can be put in a print. Learn to interpret negatives. Metal in teeth show (white) in the negative. Cement shows lighter than tooth tissue. Porcelain and silicate cement are somewhat like tooth tissue. Guttapercha stops the ray more than tooth tissue and therefore shows lighter. Enamel is denser and shows lighter than dentine inside it. The cellular structure of alveolar bone is shown beautifully. Soft gum tissue shows little, if any. The pericementum should be shown as a dark line against the root. This normally is plainer toward the neck of the tooth but should be followed about the apex of the root. Just outside the pericementum look for a light line which is the bony wall of the root socket and is shown by this light line to be compact bone. Let me emphasize the importance of looking for this light line just against and outside of the dark line made by the pericementum. This light line is never carried through a dental abscess or a granuloma. Hold your negative before an electric light strong enough to make every detail plain. The ideal light is 50 to 100 candle power with a green opal shade. Let the light shine on the film but have the shade between the light and the eyes. Surround the negative with a dark card, so the sight is not hindered by the bright light that would pass around it. Use a good magnifying glass of 10 to 15 power to see every detail. You will often follow the white socket wall toward the gingival margin and see it more or less broken into by a gingival infection. It is most interesting to study about the apical area of all dead teeth. I may offer it as a rule that whatever may appear or has been interpreted as alveolar abscess, that if the compact root socket wall can be followed about the root apex, there is no sign of apical infection. But it is comparatively rare where roots have long been dead and especially if poorly filled to find this line not broken into. I must also say that not always about healthy roots can this light line be followed. Where the root end is small and the curvature sharp, or where the tube used is of too high vacuum and too high penetration or any movement of the film during exposure, it will not be shown.

I understand that any unfilled apical portion of a dead root contains sooner or later organic matter that is suitable food for bacteria and that bacteria will get in there. Bacteria and their pockets will continually pass through the apical openings and there is war between the bacteria cells and the body cells, each trying to destroy and use the other. The body cells for better defense build around the bacteria infection a wall of granulation tissue, and this is the beginning of a granuloma. Within the dead root is no blood circulation, so no means of destroying the bacteria that never cease their invasions. Condition of the patient's health often permits the granulation wall to be frequently broken through by the invaders, necessitating a further outward growth of the granulation tissue which, in time, often leaves a more or less large socket about the root apex. The inside of the granulation, I understand, contains bacteria, that are more or less able to pass beyond the granuloma into the blood stream and may infect another part of the body for which they have an affinity or which offers too little resistance to destroy them. I can not see how an unfilled apical part of the root is ever safe, but will likely sooner or later lead to the above conditions. I know that for the past year fully half of my dental practice has been treating the mouths of people who are more or less ill supposedly from oral infections. And often I have seen people relieved by dental treatment so promptly that it has reminded me of the old days of miracles.

The radiographer is often appealed to in cases of acute abscess. And if the infection be of short duration the negative may show little or nothing abnormal. The fact has been that a violent invasion of the apical area has produced first an increased blood supply, then stasis with consequent inflammatory products extending a considerable distance about the root apex. This condition may exist about a considerable area and not more acutely at the root apex than a short distance away from it. There may follow some necroses about the apex, not often. If there does, then pus would continue to flow freely. The seguestrum of alveolar bone would break into irregular masses. The living bone about the necrosed area would be jagged and irregular. The wall outside a granuloma usually presents a rounded regular outline. The patient may never have felt the presence of a granuloma which may be a cause of almost any illness.

Cotton in a root canal never shows. A paste in cotton may have somewhat the density of the tooth and make the canal difficult to show. Ofter a guttapercha point is put in the canal to become stopped at the neck, leaving a long, thin point, often crooked, that far from fills the canal. No agent has so revealed to us the very low percentage of good root fillings as the X-ray and has so led us to make good fillings.

There is considerable difficulty in getting negatives of the upper molar regions that clearly show the parts. The negative film is placed inside the arch lying against the teeth and palate. Thus the film is not nearly in the same plane as the teeth, and if the arch is very low the film is nearly horizontal. The rule for the position of the tube is half way between a perpendicular to the teeth and a perpendicular to the film. Thus the tube must be placed high, so that the rays pass down through the antrum to get the palatal roots or to get beyond the apices of any posterior teeth. Very often the rays must pass through the malar bone or malar ridge, which is dense bone. All these structures must be taken into account in judging negatives of these parts. Often cells of the antrum show dark areas on the film like abscess areas.

The floor of the antrum always should show as a light line. Above this line we expect to find dark areas showing antrum cells. About the roots of healthy teeth shown against the antrum we may often follow the light line of the root socket wall, and inside it the dark line of the pericementum. An apical abscess as a rule lies about and against the root apex, and destroys the pericementum and socket wall. The antrum cell shows any accidental relation to the root apex.

Usually the patient holds the film in place by pressing a finger against it. This usually bends the film. The part of the film against the teeth may be nearly vertical and the part below the palate nearly horizontal. Thus the shadow of the tooth crown may be shortened and of the apical part very much lengthened. A film placed inside the centrals will likely be curved at each side, so that while the centrals may appear normal, the lateral roots may be shown too wide and cuspids very much too wide. All such conditions must be recognized in the film and judgment made accordingly. This same bending of the sides of the film is sure to occur in examining the lower central region. The floor of the nares should never be mistaken for an abscess. Everybody knows where the nose is, and the dark area of the floor of the nose is usually well above the shadow of the roots. Often, however, the end of the nose or the vomer will be shown as a shadow, say, over one central root if the rays come from one side of the center of the face. Thus the root of one central and alveolar bone about it will show much darker than the other, because the other is shadowed by the nose and vomer. Often the alveolar bone about the lateral incisor root is very thin and cancellous and the ray may be directed just at right angles to it. This often will make the part of the negative about and just above the apex of the lateral tooth so dark as to look like an abscess area. If I have any doubt of the vitality of the lateral tooth to be radiographed, I usually make that fact clear by means of an electric test to avoid any mistake in reading the negative. The areas of the lower bicuspids and molars are most easy to radiograph. The inferior dental canal and the mental foramen will likely be shown and the mental foramen is apt to appear about the end of the second bicuspid root, the apex not being in the middle of it. Even the apical end of the root within the granuloma should appear darker than the portion of the root not within the granuloma, because alveolar bone is destroyed buccally and lingually as well as mesially and distally. In the same way upper bicuspids and molar roots that overlap and thus fall in the same line of rays from the tube will be shown lighter on the film than where roots do not so overlap. This is often the only way we can trace upper molar buccal roots and the tissue about their apices.—*Oral Health.*

